# HIV Versus the Human Body: A Case Report of an Immunity-Compromised Patient

**DOI:** 10.7759/cureus.62942

**Published:** 2024-06-23

**Authors:** Ashwin Karnan, Ulhas Jadhav, Babaji Ghewade, Anjana Ledwani, Harshith Beeravolu

**Affiliations:** 1 Pulmonary Medicine, Jawaharlal Nehru Medical College, Datta Meghe Institute of Higher Education and Research, Wardha, IND; 2 Respiratory Medicine, Jawaharlal Nehru Medical College, Datta Meghe Institute of Higher Education and Research, Wardha, IND

**Keywords:** hiv/aids, lymph node tuberculosis, cd4 t-cells, immunity impairment, esophageal candidiasis, disseminated tuberculosis

## Abstract

The immune system is the body’s defense system against infection, pathogenic organisms, or foreign bodies. Human immunodeficiency virus (HIV) infection significantly reduces the number of cells involved in the immune system making the infected person prone to a greater number of infections like tuberculosis (TB). HIV infection reduces the CD4 T helper cell count and further replicates within the body. HIV-TB is a major health concern as there is more chance of progression to acquired immunodeficiency syndrome (AIDS) and the emergence of drug-resistant TB. In this case report, we see how the HIV-TB infection affects the body, significantly affecting the morbidity and mortality of the patient.

## Introduction

Immunity is the body’s ability to defend itself from foreign substances and pathogenic organisms that may enter the body. It is a complex network that primarily includes the thymus and the bone marrow and secondarily the spleen, adenoids, tonsils, and lymph nodes. The cells involved in the immune system are the phagocytes, natural killer cells, T lymphocytes, and B lymphocytes. The immune system consists of innate or nonspecific immunity and adaptive or specific immunity [1]. When there is an inborn immune deficiency, when the body’s immune system fails to attack the pathogen, or when drugs are used to suppress the body’s immune system as seen in autoimmune disease, post-organ transplantation, and diseases where there is severe inflammation, the body may be prone to a wide range of infections.

## Case presentation

A 54-year-old male, farmer, and chronic smoker presented to the outpatient department with complaints of fever, cough with mucoid expectoration, throat pain, and difficulty in swallowing for the past three months. The patient has no known comorbid conditions and gives a history of herpes zoster infection along the anterolateral aspect of the T5 and T6 dermatome one year back for which he was treated with oral antiviral drugs and analgesics.

On general examination, the patient was conscious, and oriented, grade II clubbing was present. His vitals were pulse rate of 88 beats/minute, respiratory rate of 20 breaths/minute, oxygen saturation of 96% at room air, and blood pressure of 110/60 mm hg. Respiratory system examination showed bilateral crepitations over all areas.

**Figure 1:**
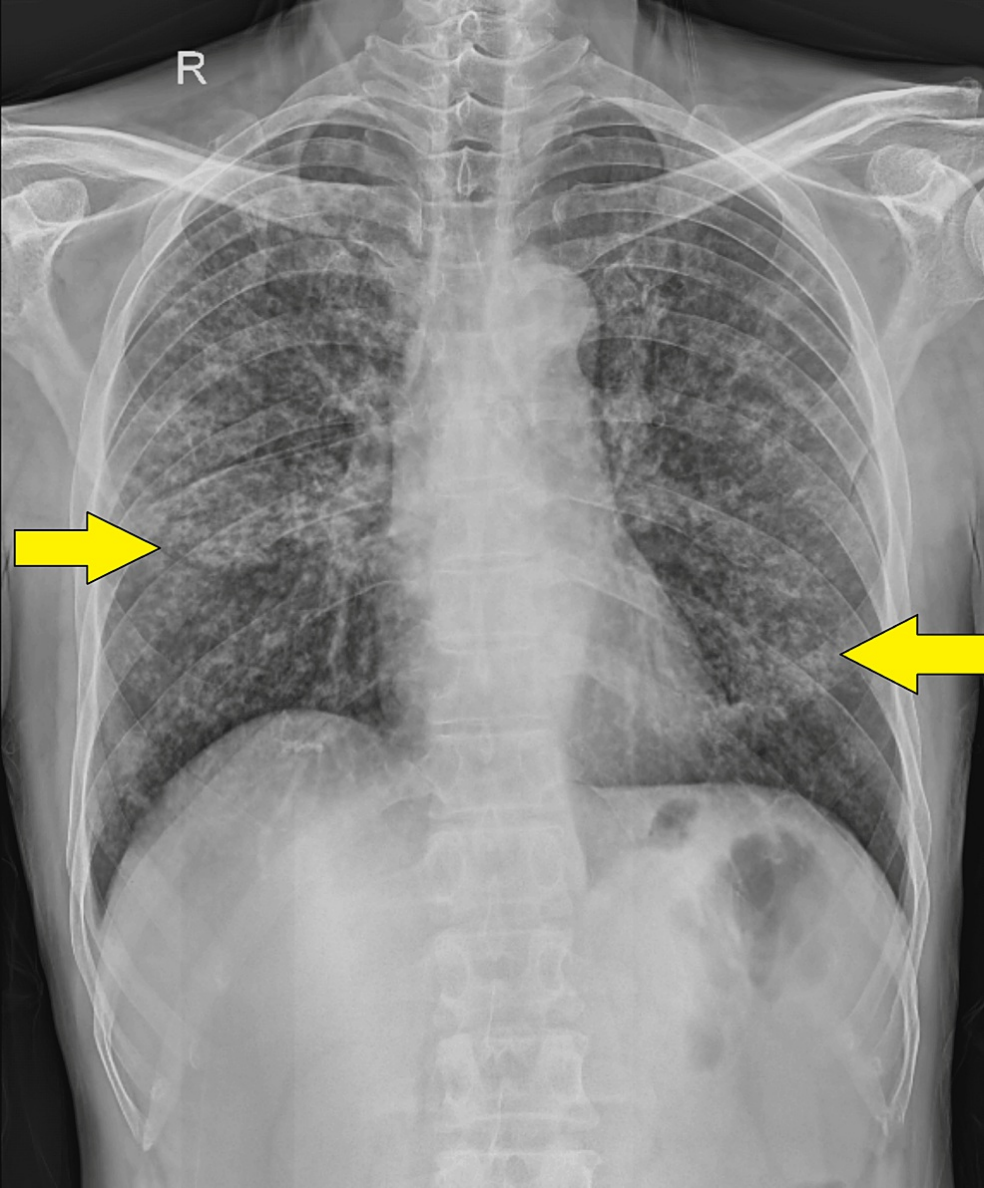
Chest X-ray of the patient showing small pulmonary nodules scattered throughout bilateral lung fields suggestive of miliary opacities.

The electrocardiogram was within normal limits. Chest X-ray (Figure 1) showed small pulmonary nodules scattered throughout bilateral lung fields suggestive of miliary opacities.

Blood investigations showed microcytic hypochromic anemia, hyponatremia, hypoalbuminemia, albumin-globulin ratio reversal, a positive nucleic acid test for human immunodeficiency virus (HIV), HIV viral load of 54,000 copies/ml, CD4 count of 198 cells/cubic mm, positive sputum for acid-fast bacilli, and isoniazid resistance in sputum for line probe assay (Table 1).

**Table 1:** Blood investigations of the patient. CD4: cluster of differentiation 4, HIV: human immunodeficiency virus

Investigation	Values	Reference range
Hemoglobin	7.8 g/dL	13.5-17.5 g/dL
Serum sodium	129 mmol/L	135-145 mmol/L
Albumin	2.4 g/dL	3.5-5.5 g/dL
Globulin	4.2 g/dL	2.0-3.5 g/dL
CD4 count	198 cells/cubic mm	500-1500 cells/cubic mm
HIV viral load	54,000 copies/mL	

**Figure 2:**
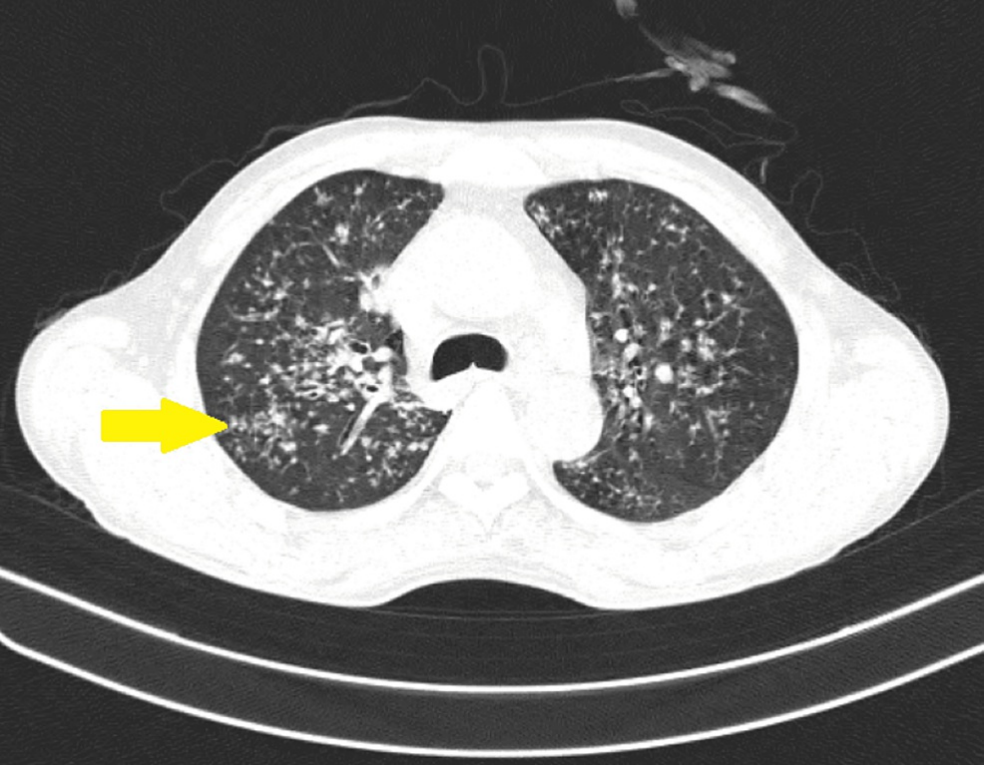
Computed tomography of the thorax showing variable-sized centrilobular nodules appearing as linear branching patterns suggestive of miliary tuberculosis.

**Figure 3:**
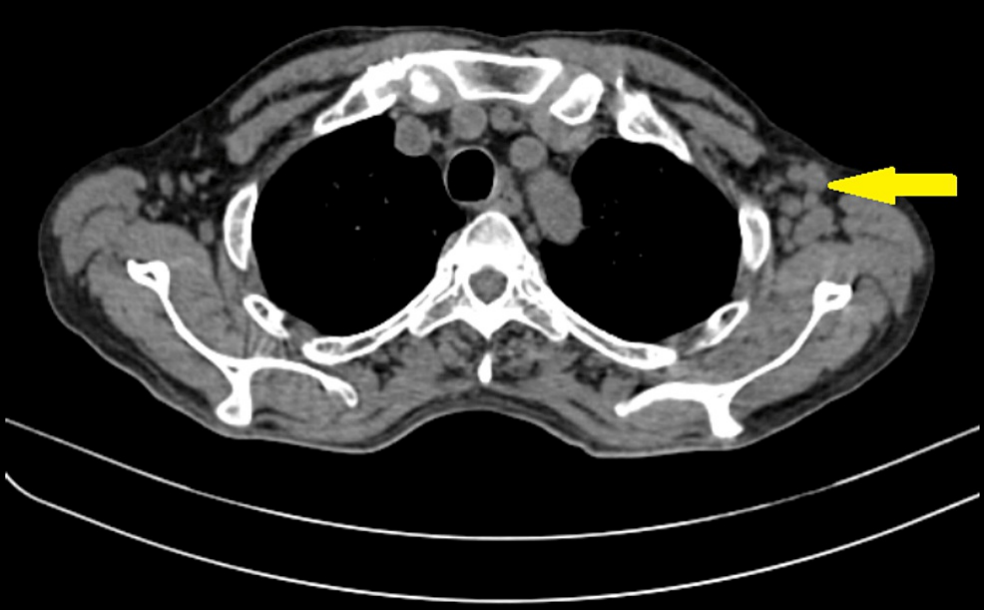
Computed tomography of the thorax, mediastinal window showing enlarged left axillary group of lymph nodes.

Contrast-enhanced computed tomography (Figures 2, 3) of the thorax and the abdomen was done, which showed multiple variable-sized centrilobular nodules appearing as linear branching patterns giving tree-in-bud appearance in bilateral lung fields with enlarged bilateral axillary and cervical lymph nodes and long-segment circumferential mild enhancing thickening in the terminal ileum, ileocaecal junction, and medial wall of caecum with multiple pre-aortic, para-aortic, retro-caval and bilateral common and external iliac lymph nodes.

**Figure 4:**
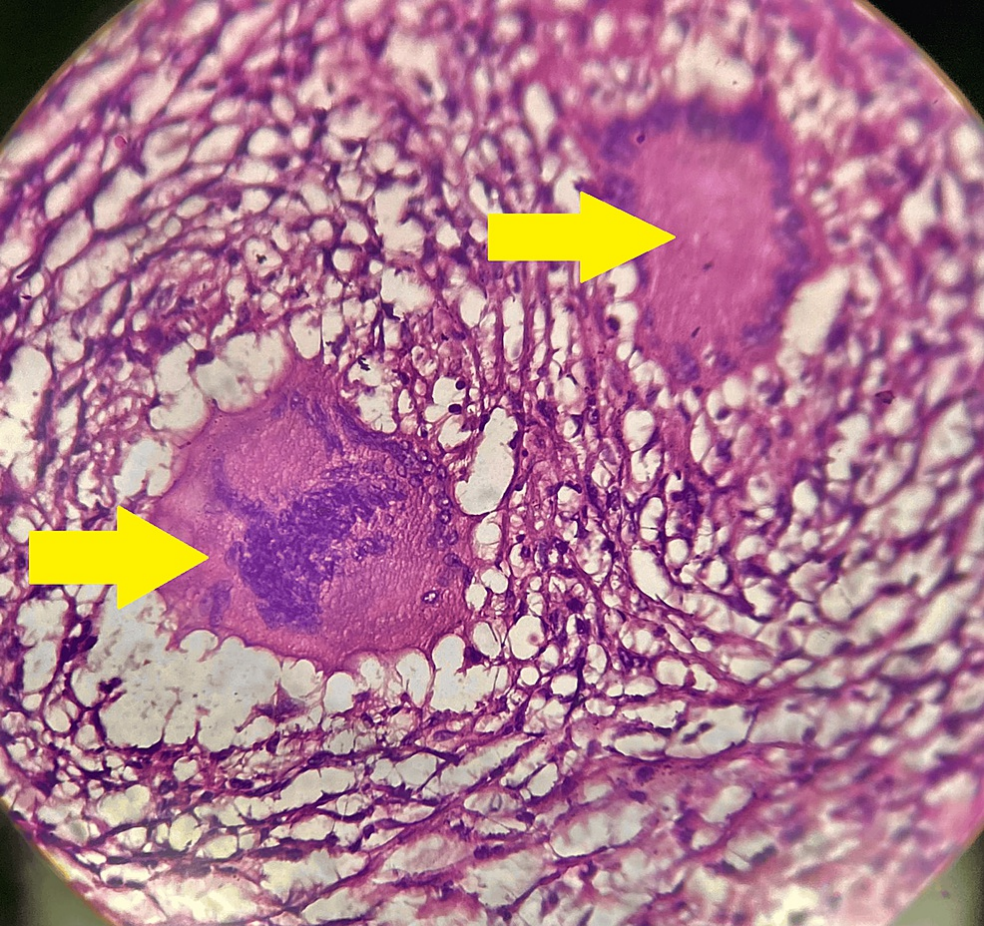
Lymph node biopsy sample showing the presence of Langhans giant cells on cytological examination.

A cervical lymph node biopsy (Figure 4) was done, which showed the presence of granuloma with giant cells and areas of caseation suggestive of tubercular etiology.

**Figure 5:**
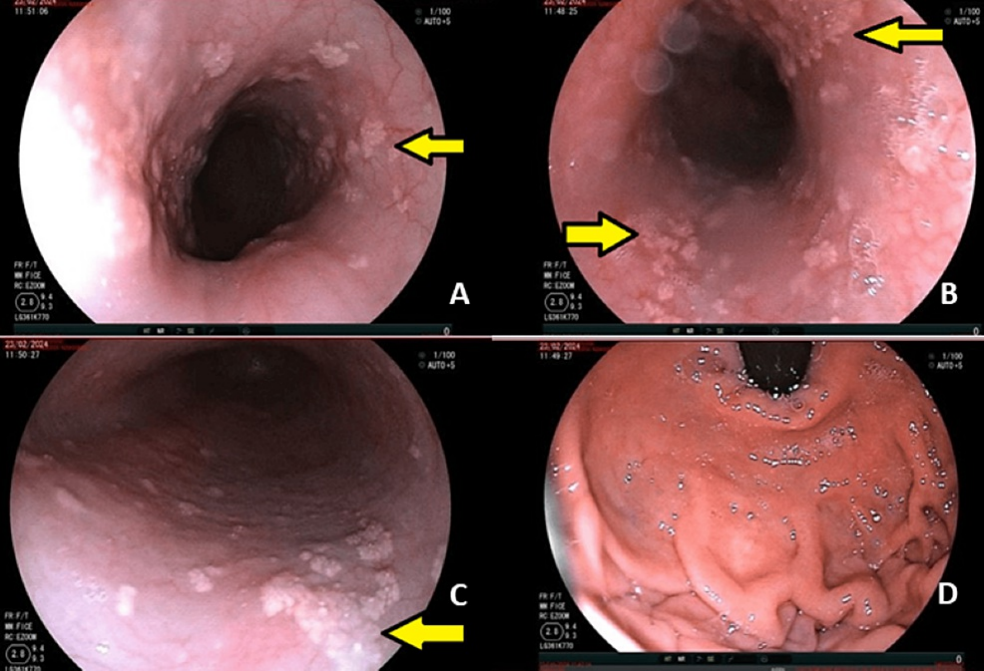
Endoscopy images of the patient showing the presence of whitish plaques on the esophageal mucosa.

After consultation with a gastroenterologist for persistent dysphagia, the patient underwent an endoscopy, which showed whitish plaques along the esophageal mucosa pointing toward esophageal candidiasis (Figure 5).

During the hospital stay, the patient developed multiple episodes of loose stools. Initially, the patient was suspected to have pseudomembranous colitis. Due to the non-resolution of symptoms, everyday stool samples for consecutive four days were sent for routine examination and culture, which showed the presence of *Cryptosporidium* under direct microscopy in one sample by the modified acid-fast staining procedure. Molecular tests and enzyme assays could not be done due to the unavailability of the test in the laboratory.

The patient was treated with weight-based anti-tubercular drugs according to the isoniazid monoresistance regimen, intravenous broad-spectrum antibiotic piperacillin with tazobactam, intravenous fluconazole for 14 days for candidiasis, azithromycin for five days for cryptosporidium diarrhea, prophylactic trimethoprim with sulfamethoxazole, diphenoxylate, iron supplements, intravenous fluids, nutritional high-protein diet, and physiotherapy. Oral corticosteroid used by many in miliary tuberculosis (TB) was kept on standby and not used in this patient. A liver function test was monitored throughout the hospital stay to look for possible drug-induced hepatitis. The patient improved symptomatically and was discharged and is currently under follow-up. Antiretroviral therapy was planned to be started at six weeks after discharge.

## Discussion

HIV is a retrovirus that causes HIV infection, which in the later stages leads to acquired immune deficiency syndrome (AIDS) [2]. According to the 2022 Global World Health Organization (WHO) HIV report, an estimated 39 million people are living with HIV and around 630,000 people died due to HIV in 2022 [3]. HIV infection is spread majorly by unprotected intercourse, through the sharing of contaminated needles, infected blood transfusions due to improper screening, and during pregnancy from an infected mother to fetus. The HIV mainly targets the CD4 cells and macrophages. Two major forms are recognized, HIV-1 and HIV-2. There are three stages of HIV infection [4]: Stage 1 is an acute infective stage where within two to four weeks after infection with HIV, the patient develops fever, headache, rashes, and generalized tiredness. During this stage, the viral load in the blood is significantly high. Stage 2 is a chronic infective stage or stage of latency or asymptomatic stage where the patient has no significant complaints but the virus continues to replicate within the body. Years after the chronic infective stage, the patient progresses to AIDS which is Stage 3, where the CD4 count in the body is significantly reduced to levels <200 cells/cubic mm, making the patient prone to many opportunistic infections.

Depending upon the CD4 count, the following opportunistic infections are seen (Table 2) [5].

**Table 2:** Opportunistic infection seen according to the CD4 cell count in blood. HIV: human immunodeficiency virus, CMV: cytomegalovirus. Citation: Singh et al. [5]

CD4 count( cells/cubic mm)	Opportunistic infections
<500	Tuberculosis, herpes zoster, lymphoma, pneumonia, candidiasis
<200	Pneumocystis pneumonia, Isospora diarrhea, HIV wasting syndrome, HIV dementia
<100	Toxoplasmosis, cryptococcal meningitis, cytomegalovirus (CMV) infection, Mycobacterium avium complex
<50	CMV infections, Toxoplasmodium gondi, Cryptosporidium

The treatment for HIV infection warrants starting antiretroviral therapy (ART) as soon as possible. The regimen includes a combination of a nucleoside reverse transcriptase inhibitor, a non-nucleoside reverse transcriptase inhibitor, a protease inhibitor, or an integrase inhibitor. Currently, combination pills are available for better patient compliance due to once-daily administration. The treatment for HIV infection is a multisystemic approach requiring an interdepartmental approach of chemotherapy, physiotherapy, nutrition, counseling, regular follow-up, and preventing the spread of the infection to another person.

TB is a major public health issue caused by *Mycobacterium tuberculosis*. The prevalence is about 31.3%, with about 10.6 million new cases reported in 2021 according to the Global TB Report 2022. The WHO End TB strategy aims to reduce TB deaths and reduce 90% of new TB cases by 2035 [6]. Disseminated TB is the term used to describe the lympho-hematogenous spread of *Mycobacterium tuberculosis* involving two noncontiguous sites. It is a life-threatening condition, accounting for around 2% of all TB cases and 20% of extrapulmonary TB in immunocompetent individuals [7]. Miliary TB is a form of disseminated TB resulting from a massive hematogenous spread of MTB characterized by small nodular, millet-sized shadows on chest X-ray. It accounts for almost 50% of extrapulmonary TB in immunosuppressed individuals [8]. It has a male predominance both in pediatric as well as adult groups. Miliary TB can occur at the time of primary infection as a result of primary progressive TB due to hematogenous spread or due to reinfection or reactivation of a dormant focus. The predisposing conditions to miliary TB are listed in Table 3.

**Table 3:** Predisposing conditions to miliary tuberculosis. HIV: human immunodeficiency virus, AIDS: acquired immunodeficiency syndrome. Citation: Sharma and Mohan [8]

Predisposing conditions to miliary tuberculosis
1. HIV/AIDS
2. Diabetes mellitus
3. Chronic kidney disease
4. Chronic alcoholism
5. Malnutrition
6. Underlying malignancy
7. Post solid organ transplantation
8. Childhood infections
9. Immunosuppressant drugs

Tuberculin skin test, interferon-gamma release assay, sputum examination, bronchoalveolar lavage, chest radiograph, adenosine deaminase levels, cerebrospinal fluid examination, fine-needle aspiration cytology of lymph nodes, ascetic fluid examination, pleural fluid examination, and high-resolution computed tomography are some of the investigations, which may aid in the diagnosis of miliary TB. Treatment with anti-tubercular drugs is the cornerstone of management. Since TB is associated with adrenal insufficiency, corticosteroids play an important role. They are also indicated in cases of TB meningitis, immune reconstitution inflammatory syndrome (IRIS), pleural effusion, pericardial effusion, and immune complex nephritis to reduce inflammation.

In the majority of people infected with *Mycobacterium tuberculosis*, they do not develop an active infection. HIV co-infection is an independent risk factor for developing active TB. The immune suppression in AIDS is the loss of CD4 cells in blood and other areas making them prone to active TB infection as CD4 cells are known to stimulate alveolar macrophages, which engulf the TB bacilli. TB also exacerbates the progression of HIV infection to AIDS. When treating patients infected with HIV and TB, one must always keep in mind IRIS, which is an abnormal exacerbated immunological response to *Mycobacterium* [9]. There are two types of IRIS, paradoxical IRIS and unmasking IRIS. Low CD4 count before antiretroviral treatment initiation leading to a rise in their count is one of the major risk factors for developing IRIS. Symptomatic management is the treatment of choice, but in IRIS of the central nervous system, high-dose corticosteroids may be needed. To prevent IRIS and drug interactions, HIV and TB treatment may be initiated accordingly, i.e., CD4 count less than 50 cells/cubic mm (initiate ART within two weeks of anti-tubercular therapy (ATT)) and CD4 count more than 50 cells/cubic mm (initiate ART eight weeks after ATT) [10].

*Candida* is a type of fungi that is a part of the normal flora of the digestive and urinary tract. When there is an impairment of the immune system, candidiasis may occur. The oropharynx followed by the esophagus is the common site of *Candida* infection in the gastrointestinal system [11]. Patients with HIV, diabetes, and leukemia, smokers, and patients on steroids and chemotherapy are prone to infection. Diagnosis is by endoscopy where the esophageal mucosa shows whitish plaque or ulceration and on histology pseudohyphae may be seen. Treatment is by oral or intravenous fluconazole for two weeks. Other treatment options include itraconazole and voriconazole, and in severe cases, amphotericin B may be indicated.

*Cryptosporidium* is a protozoon that causes diarrhea by affecting the brush border layer of the intestine also leading to malabsorption. It is spread by fecal-oral transmission through contaminated water and also through exposure to cats and cattle [12]. After ingestion, sporozoites are released in the small intestine where they multiply massively and also shed oocysts which may be found in stool. Diagnosis is by stool examination for mature oocysts, antigen assays, and molecular testing via polymerase chain reaction. Nitazoxanide and azithromycin may be used but the mainstay is immune reconstitution.

Herpes zoster is a viral disease caused by reactivation of the Varicella zoster virus, which remains dormant in the sensory ganglia of the cranial nerve after primary infection. The incidence ranges from 3.9 to 12 per 1000 people in those aged over 65 years [13]. Clinically, the dermatologic involvement is centripetal and follows a dermatome, which initially begins as erythematous papules and later becomes vesicles. The three stages of infection are the pre-eruptive stage, acute eruptive stage, and chronic infective stage. Antiviral therapy with acyclovir, valacyclovir, or famciclovir along with analgesic and topical creams are used. 

Clubbing is a sign seen as bulbous enlargement of one or more fingers or toes due to obliteration of the angle between the nail and the nailbed [14]. It is painless and may be unilateral, unidigital, or bilateral. Various hypotheses are involved behind the development of clubbing, which include neurocirculatory reflex, chronic hypoxia, platelet-derived growth factor (PDGF), and vascular endothelial growth factor causing vasodilation. According to studies, there is a 30% incidence of clubbing in patients infected with TB and HIV infections [15].

## Conclusions

HIV infection can be detrimental, thereby significantly reducing the lifespan of a patient. As the disease progresses, the weakening immune system makes the patient prone to multiple opportunistic infections. The prognosis of opportunistic infections in HIV patients remains poor. Once antiretroviral therapy is started, the viral load reduces, and the CD4 T cell count improves, resulting in immune restoration and a significant reduction in morbidity and mortality. Beyond infection, HIV is known to cause lymphoma, malignancy, pulmonary arterial hypertension, wasting syndrome, nephropathy, and thromboembolic disorders. Hence, a multidisciplinary approach is to be planned right from the cessation of substance addiction, which includes early diagnosis; detailed counseling about the nature, course, and prognosis of the disease; proper treatment regimen; lifestyle modification; protein-rich diet; tackling the social stigma of HIV; and need for a regular checkup and follow-up. Moreover, government organizations must provide financial support if needed.
